# Compilation of longitudinal gut microbiome, serum metabolome, and clinical data in acute myeloid leukemia

**DOI:** 10.1038/s41597-022-01600-2

**Published:** 2022-08-02

**Authors:** Armin Rashidi, Maryam Ebadi, Tauseef Ur Rehman, Heba Elhusseini, Hossam Halaweish, Thomas Kaiser, Shernan G. Holtan, Alexander Khoruts, Daniel J. Weisdorf, Christopher Staley

**Affiliations:** 1 Division of Hematology, Oncology, and Transplantation, Department of Medicine, University of Minnesota, Minneapolis, MN USA; 2 Department of Surgery, University of Minnesota, Minneapolis, MN USA; 3 Division of Gastroenterology, Hepatology, and Nutrition, Department of Medicine, University of Minnesota, Minneapolis, MN USA

**Keywords:** Microbiome, Acute myeloid leukaemia

## Abstract

Induction chemotherapy for patients with acute myeloid leukemia (AML) is a unique clinical scenario. These patients spend several weeks in the hospital, receiving multiple antibiotics, experiencing gastrointestinal mucosal damage, and suffering severe impairments in their immune system and nutrition. These factors cause major disruptions to the gut microbiota to a level rarely seen in other clinical conditions. Thus, the study of the gut microbiota in these patients can reveal novel aspects of microbiota-host relationships. When combined with the circulating metabolome, such studies could shed light on gut microbiota contribution to circulating metabolites. Collectively, gut microbiota and circulating metabolome are known to regulate host physiology. We have previously deposited amplicon sequences from 566 fecal samples from 68 AML patients. Here, we provide sample-level details and a link, using de-identified patient IDs, to additional data including serum metabolomics (260 samples from 36 patients) and clinical metadata. The detailed information provided enables comprehensive multi-omics analysis. We validate the technical quality of these data through 3 examples and demonstrate a method for integrated analysis.

## Abstract (summary)

**Table:** 

Measurement(s)	Short amplicon sequence variant (ASV) abundances in human stool • Metabolite abundances in human serum
Technology Type(s)	Bacterial 16 S RNA • liquid chromatography-mass spectrometry
Sample Characteristic - Organism	Bacteria
Sample Characteristic - Environment	feces

## Background & Summary

The standard curative-intent chemotherapy for patients with acute myeloid leukemia (AML) is accompanied by major intestinal microbiota disruptions due mainly to high antibiotic pressure during several weeks of hospitalization^1–3^. The impact of dysbiosis in these patients has only partially been characterized and includes neutropenic fever^4^, bloodstream infection^3,5^, and increased mortality^6^. The involved mechanisms are even less clear and include intestinal domination^6^, gut barrier damage^5^, and altered microbiota-host crosstalk^4^. One of the reasons for our poor understanding of how dysbiosis may influence clinical outcomes in these patients is the inherently high-risk nature of mechanistic studies in this patient population. Intensive chemotherapy suppresses the bone marrow and the resulting decline in platelets and white blood cells make safe access to the intestinal tract and associated interventions challenging and often infeasible due to high bleeding and infection risk. The period of neutropenia typically starts during week 1 of chemotherapy and, in patients achieving a complete remission, ends in week 4.

With these limitations in mind, a good alternative strategy to achieve mechanistic knowledge is by analyzing longitudinal datasets of multi-omic data. Multi-omics data assess different but related aspects of pathogenesis which are often on a causal link. For example, the gut microbiota regulates and significantly contributes to the circulating metabolome^7–9^. In addition, longitudinal data provide insight into intra-individual patterns of change over time^10^ which should be distinguished from inter-individual variation due to the personalized features of the microbiota and relevant host-specific factors^11^. Knowledge obtained from longitudinal multi-omics studies facilitates the generation of testable hypotheses for subsequent therapeutic trials^12^.

Here, we provide patient- and sample-level longitudinal gut microbiota and circulating microbiome data from AML patients hospitalized to receive induction chemotherapy at the University of Minnesota. Since the initiation of our effort to collect this biorepository, we have been depositing the raw 16S amplicon sequences from the stool samples in the form of paired-end fastq files at the NCBI’s Sequence Read Archive^13^. In addition, we recently analyzed a subset of longitudinal serum samples obtained from the same patients for metabolomics and reported them in aggregate^4^. Here, we provide patient- and sample-level metabolomic data and a link between serum and stool samples for each patient. In addition, we provide granular antibiotic exposure data (facilitated by the patients’ hospitalized and their closely monitored status) and other clinical metadata for each patient. Collectively, the user will have access to the longitudinal gut microbiome, serum metabolome, and clinical metadata of each patient. This unique database will enable hypothesis generation about gut microbiota and circulating metabolomic changes within and between individuals over time, their possible causal connections, and how baseline and subsequent clinical factors may influence the microbiome and metabolome. Prolonged hospitalization, heavy antibiotic exposure to prevent and treat infections, severe decline in the immune system, cytotoxic damage to the intestinal barrier, and nutritional compromise make the patient population of interest in this study unique. Therefore, we expect the well-annotated multi-omics data compiled here to lead to new discoveries in humans experiencing severe multi-faceted perturbations.

## Methods

### Participants and clinical metadata

Sample collection and analysis was approved by the University of Minnesota Institutional Review Board (ClinicalTrials.gov Identifier: NCT03316456). All participants provided signed informed consent. Clinical metadata was obtained by reviewing the electronic medical records. In the first step, 3 of the investigators independently collected data. In the second step, a fourth investigator compared the findings between the reviewers and resolved any conflicts. All identifiable data were removed. Patient_ID is a non-identifiable indicator that can be used to link clinical metadata to omics sample data. All dates are relative to the first day of chemotherapy, which itself will remain confidential. This protocol was initiated in 2017 and closed in 2021. This article includes all data from the study.

### Sample collection and fecal 16S rRNA gene sequencing

Serum and stool sample collection started with hospital admission and continued twice weekly (Mon/Thu + /− 2 days) until day 28 of chemotherapy or discharge (whichever occurred first). Serum samples were collected twice weekly (Mon/Thu; preprandial) between 6–8 AM in standard red-top tubes, split in 250 μL aliquots, and stored at −80 °C within 2 hours of collection. Stool samples were collected in 95% ethanol-filled sterile tubes and stored at −80 °C. DNA from the stool samples was extracted using the DNeasy PowerSoil DNA isolation kit (QIAGEN, Hilden, Germany). qPCR was used to quantify 16S rRNA gene content in each sample. The V4 hypervariable region of the 16S rRNA gene was amplified on an Illumina MiSeq platform (2 × 300 paired-end mode)^14^. Adaptor trimming was done using SHI7^15^, and the resulting demultiplexed fastq files were used as input to DADA2^16^ to infer exact amplicon sequence variants (ASVs) (*dada2* package v1.18.0 in R). For filtering, we used DADA2 default parameters (PHRED score threshold of 2, maximum number of expected errors of 2 for both forward and reverse reads) and truncation lengths of 220 (forward) and 150 (reverse). De-replication, de-noising, merging, and chimera removal were done using DADA2 default parameters. Taxonomic assignment was done by the naive Bayesian classifier implemented in DADA2 and the SILVA non-redundant v138.1 training set^17^. Clinical metadata and ASV abundances were merged into a phyloseq object in R (R Foundation for Statistical Computing, Vienna, Austria) for analysis.

### Serum metabolome profiling

Serum samples were sent to Metabolon (Morrisville, NC) for untargeted, ultrahigh performance liquid chromatography-tandem mass spectroscopy (UPLC-MS/MS).

#### Sample preparation

Samples were prepared using the automated MicroLab STAR® system from Hamilton Company. A total of 100 μL of sample was extracted under vigorous shaking for 2 min (Glen Mills GenoGrinder 2000) with methanol 80%, containing the following recovery standards: DL-2-fluorophenylglycine, tridecanoic acid, d6-cholesterol, and DL-4-chlorophenylalanine. The resulting extract was divided into 5 fractions: two for analysis by two separate reverse phase (RP)/UPLC-MS/MS methods with positive ion mode electrospray ionization (ESI), one for analysis by RP/UPLC-MS/MS with negative ion mode ESI, and one for analysis by HILIC/UPLC-MS/MS with negative ion mode ESI. The remaining aliquot was reserved for backup. Samples were placed briefly on a TurboVap® (Zymark) to remove the organic solvent. The sample extracts were stored overnight under nitrogen before preparation for analysis.

#### Mass spectrometry

All methods utilized a Waters ACQUITY UPLC and a Thermo Scientific Q-Exactive high-resolution/accurate mass spectrometer interfaced with a heated electrospray ionization (HESI-II) source and Orbitrap mass analyzer operated at *R* = 35,000 mass resolution. The sample extract was dried then reconstituted in solvents compatible to each of the four methods. For each sample, two aliquots of each sample were reconstituted in 50 μL of 6.5 mM ammonium bicarbonate in water (pH 8) for the negative ion analysis and another two aliquots of each were reconstituted using 50 μL 0.1% formic acid in water (pH ~3.5) for the positive ion method. Each reconstitution solvent contained a series of standards at fixed concentrations to ensure injection and chromatographic consistency. The internal standards consist of a variety of deuterium labeled or halogenated biochemicals specifically designed both to cover the entire chromatographic run and to not interfere with the detection of any endogenous biochemicals. Authentic standards of d7-glucose, d3-leucine, d8-phenylalanine, and d5-tryptophan were purchased from Cambridge Isotope Laboratories (Andover, MA). d5-hippuric acid, d5-indole acetic acid, and d9-progesterone were procured from C/D/N Isotopes, Inc. (Pointe-Claire, Quebec). Bromophenylalanine was provided by Sigma-Aldrich Co. LLC. (St. Louis, MO) and amitriptyline was from MP Biomedicals, LLC. (Aurora, OH). Recovery standards of DL-2-fluorophenylglycine and DL-4-chlorophenylalanine were from Aldrich Chemical Co. (Milwaukee, WI). Tridecanoic acid was purchased from Sigma-Aldrich (St. Louis, MO) and d6-cholesterol was from Cambridge Isotope Laboratories (Andover, MA). Standards for the HILIC dilution series of alpha-ketoglutarate, ATP, malic acid, NADH, and oxaloacetic acid were purchased from Sigma-Aldrich Co. LLC. (St. Louis, MO) while succinic acid, pyruvic acid and NAD + were purchased from MP Biomedicals, LLC. (Santa Ana, CA). Limit of detection (LOD) for standards analyzed in a dilution series using reverse phase chromatography is available in Table 1.

**Table 1:** Limit of detection (LOD) for standards in a dilution series using reverse-phase chromatography.

Standard	HRAM LOD ng/mL	UMR LOD ng/mL
d7-glucose	1.0	50.0
d3-leucine	0.25	5.0
d8-phenylalanine	0.25	3.0
d5-tryptophan	0.25	25.0
d5-hippuric acid	0.25	5.0
Br-phenylalanine	0.25	3.0
d5-indole acetic acid	3.0	25.0
amitriptyline	0.5	3.0
d9-progesterone	1.0	25.0

One aliquot was analyzed using acidic positive ion conditions (LC pos), chromatographically optimized for more hydrophilic compounds. In this method, the extract was gradient eluted from a C18 column (Waters UPLC BEH C18-2.1 × 100 mm, 1.7 µm) using water and methanol, containing 0.05% perfluoropentanoic acid (PFPA) and 0.1% formic acid (FA) at pH = 2.5. Elution was performed at 0.35 mL min−1 in a linear gradient from 5% to 80% of methanol containing 0.1% FA and 0.05% PFPA over 3.35 min. A second aliquot was also analyzed using acidic positive ion conditions; however, it was chromatographically optimized for more hydrophobic compounds. In this method, the extract was gradient eluted from the same aforementioned C18 column using methanol 50%, acetonitrile 50%, water, 0.05% PFPA, and 0.01% FA at pH = 2.5 and was operated at an overall higher organic content. Elution was performed at 0.60 mL/min in a linear gradient from 40% to 99.5% over 1 min, hold 2.4 min at 99.5% of methanol 50%, acetonitrile 50%, 0.05% PFPA, and 0.01% FA. A third aliquot was analyzed using basic negative ion-optimized conditions with a separate dedicated C18 column (LC neg). The basic extracts were gradient eluted from the column using methanol 95% and water 5%, with 6.5 mM ammonium bicarbonate at pH 8. Elution was performed at 0.35 mL min−1 with a linear gradient from 0.5% to 70% of methanol 95%, water 5% with 6.5 mM ammonium bicarbonate over 4 min, followed by a rapid gradient to 99% in 0.5 min. The sample injection volume was 5 μL and a 2 × needle loop overfill was used. Separations utilized separate acid and base-dedicated 2.1 mm × 100 mm Waters BEH C18 1.7 μm columns held at 40 °C. The fourth aliquot was analyzed via negative ionization following elution from an HILIC column (LC HILIC) (Waters UPLC BEH Amide 2.1 × 150 mm, 1.7 µm, held at 40 °C) using a gradient consisting of water (15%), methanol (5%), and acetonitrile (80%) with 10 mM ammonium formate, pH 10.16. Elution flow rate was 0.5 mL/min with a linear gradient from 5% to 50% in 3.5 min, followed by a linear gradient from 50% to 95% in 2 min, of water (50%), acetonitrile (50%) with 10 mM ammonium formate, pH 10.6. The MS analysis alternated between MS and data-dependent MSn scans using dynamic exclusion. The scan range varied slightly between methods but covered 70–1000 *m/z*.

#### Quality assurance and quality control (QA/QC)

**Fig. 1:**
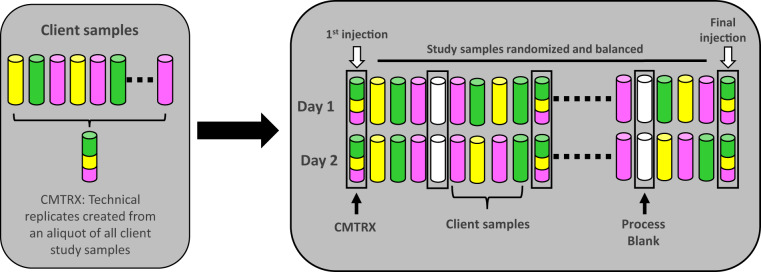
Preparation of client-specific technical replicates. A small aliquot of each sample (colored cylinders) is pooled to create a CMTRX technical replicate sample (multi-colored cylinder), which is then injected periodically throughout the platform run. Variability among consistently detected biochemicals can be used to calculate an estimate of overall process and platform variability.

Several types of controls were analyzed in concert with the experimental samples: a pooled matrix sample generated by taking a small volume of each experimental sample served as a technical replicate throughout the platform run; extracted water samples served as process blanks; and a cocktail of QC standards (carefully chosen not to interfere with the measurement of endogenous compounds) spiked into every analyzed sample allowed instrument performance monitoring and aided chromatographic alignment. Tables 2–4 describe QC samples and standards. Instrument variability was determined by calculating the median relative standard deviation (RSD) for the internal standards that were added to each sample prior to injection into the mass spectrometers (median RSD = 3–4%). Instruments are calibrated at least weekly in the utilized polarity using thermo and mass accuracy is monitored at the batch level for the internal standards. A batch fails QC if any of the internal standards are more than 5 ppm away from the theoretical mass. Overall process variability was determined by calculating the median RSD for all endogenous metabolites (i.e., non-instrument standards) present in 100% of the pooled matrix (MTRX) samples, which are technical replicates created from a large pool of extensively characterized human plasma. The median RSD for MTRX samples was 9–10%. Five MTRX samples and three process blank samples were processed per every batch of 30 samples. Experimental samples were randomized across the platform run with QC samples spaced evenly among the injections, as outlined in Fig. 1. All studies include the analysis of a technical replicate of a sample pooled from the experimental samples. This pool was analyzed 16 times over the course of the analysis of the experimental samples in the present study.

**Table 2:** Description of metabolon QC samples.

Type	Description	Purpose
CMTRX	Pool created by taking a small aliquot from every customer samples.	Assess the effect of a non-plasma matrix on the Metabolon process and distinguish biological variability from process variability.
PRCS	Aliquot of ultra-pure water	Process Blank used to assess the contribution to compound signals from the process.
SOLV	Aliquot of solvents used in extraction.	Solvent Blank used to segregate contamination sources in the extraction.

**Table 3:** Metabolon QC standards.

Type	Description	Purpose
RS	Recovery Standard	Assess variability and verify performance of extraction and instrumentation.
IS	Internal Standard	Assess variability and performance of instrument.

**Table 4:** Quality control internal standards.

Condition	Internal standards
LC neg	d7-glucose
	d3-methionine
	d3-leucine
	d8-phenylalanine
	d5-tryptophan
	Br-phenylalanine
	d15-octanoic acid
	d19-decanoic acid
	d27-tetradecanoic acid
	d35-octadecanoic acid
	d2-eicosanoic acid
LC HILIC	d35-octadecanoic acid
	d5-indole acetic acid
	Br-phenylalanine
	d5-tryptophan
	d4-tyrosine
	d3-serine
	d3-aspartic acid
	d7-ornithine
	d4-lysine
LC pos	d7-glucose
	d3-methionine
	d3-leucine
	d8-phenylalanine
	d5-tryptophan
	Br-phenylalanine
	d4-tyrosine
	d5-indole acetic acid
	d5-hippuric acid
	amitriptyline
	d9-progesterone
	d4-dioctylphthalate

#### Compound identification and quantification

Raw data were extracted, peak-identified, and QC processed using Metabolon pipelines. Compounds were identified by comparison to library entries of purified standards or recurrent unknown entities^18,19^. Briefly, Metabolon maintains a library based on authenticated standards (analyzed using the same methodology as the experimental samples) that contains the retention time/index (RI), mass to charge ratio (*m/z)*, and chromatographic data (including MS/MS spectral data) on all molecules present in the library. Furthermore, biochemical identifications are based on three criteria: retention index within a narrow RI window of the proposed identification (typically within a 5 second window), accurate mass match to the library ± 10 ppm (typically well within a 5 ppm window), and the MS/MS forward and reverse scores between the experimental data and authentic standards. The MS/MS scores are based on a comparison of the ions present in the experimental spectrum to the ions present in the library spectrum. While there may be similarities between these molecules based on one of these factors, the use of all three data points can be utilized to distinguish and differentiate biochemicals. The QC and curation processes were designed to ensure accurate and consistent identification of true chemical entities, and to remove those representing system artifacts, mis-assignments, and background noise. Metabolon data analysts use proprietary visualization and interpretation software to confirm the consistency of peak identification among the various samples. Library matches for each compound were checked for each sample and corrected if necessary. Peaks were quantified using area-under-the-curve. A data normalization step was performed to correct variation resulting from instrument inter-day tuning differences. Essentially, each compound was corrected in run-day blocks by registering the medians to equal 1 and normalizing each data point proportionately. Metabolites were assigned to pathways based on three publicly available key chemical information resources: PubChem, HMDB and KEGG pathway database.

## Data Records

Raw sequences from the stool samples were deposited at the NCBI’s Sequence Read Archive^13^. The following data were compiled and deposited in Figshare^20^:

MetabolitesIDTable: A table containing all detected serum metabolites

- Metabolite_ID: metabolite identifier
- Metabolite: scientific name of the metabolite. Compounds for which no authentic standards were available for confirmation are marked with an “*” after the compound name to designate these as Metabolomics Standards Initiative level 2/3^21^. All other metabolites were identified at level 1. The Metabolomics Standards Initiative has provided a consensus classification and notation for the level of confidence in metabolite identification. Level 1 indicates the highest level of confidence in the identity of the compound where at least 2 orthogonal properties of an authentic chemical standard are compared to experimental data acquired in the same laboratory with the same analytical methods. More than 3300 commercially available purified standard compounds have been acquired by Metabolon and registered for analysis on all platforms for determination of their analytical characteristics. Levels 2 and 3 indicate reasonable confidence in metabolite identification despite lack of an authentic standard. Specifically, level 2 (putative identification) reveals probable structure using fragmentation data from literature and/or libraries and databases, while level 3 (tentative structural identification) includes a unique match with data searched through literature and/or libraries and databases. Most such identifications are based on the experimental signature having the same characteristics as the compound class. For example, sphingomyelins all have a conserved fragmentation spectrum and so have a highly diagnostic pattern in order to permit the identification of the experimental signature as a sphingomyelin.
- SuperPathway: superpathway containing the metabolite
- SubPathway: subpathway containing the metabolite
- HMDB: The Human Metabolome Database identifier for the metabolite^22^
- KEGG: The Kyoto Encyclopedia of Genes and Genomes identifier for the metabolite^23^
- PUBCHEM: PubChem identifier for the metabolite^24^

AntibioticsTable: A table containing antibacterial antibiotic exposure data between hospital admission and day 28 of chemotherapy or discharge (whichever occurred first)

- Patient_ID
- AntibacterialABx: antibiotic name
- StartDayRelativeToD1Chemo: initiation date of the antibiotic relative to day 1 of chemotherapy
- StopDayRelativeToD1Chemo: end date of the antibiotic relative to day 1 of chemotherapy
- Route: route of administration

SerumMetabolitesRawTable: A table containing metabolite levels in serum samples (260 samples from 36 patients)

- Patient_ID
- SampleDayRelativeToD1Chemo: serum sample collection date relative to day 1 of chemotherapy
- Columns C and later: raw metabolite levels, with column name corresponding to Metabolite_ID in the MetabolitesIDTable file

SerumMetabolitesNormalizedTable: A table containing batch normalized (by median) metabolite levels in SerumMetabolitesRawTable

StoolSamplesTable: A table containing stool sample data (566 samples from 68 patients)

- Accession_Number: SRR accession number for each sample
- Patient_ID
- SampleDayRelativeToD1Chemo: stool sample collection date relative to day 1 of chemotherapy
- Quality: quality control data. A minimum threshold of 1000 copies/mL of 16S rRNA gene quantified by qPCR is considered adequate by the University of Minnesota Genomics Center. Smaller values should be treated with caution.

PatientMetadata: A table containing patient metadata (68 patients)

- Patient_ID
- Age
- Sex
- Disease_Phase: disease phase
- Chemotherapy: induction chemotherapy regimen.
- FirstNFDayRelativeToD1Chemo: day of the first neutropenic fever relative to day 1 of chemotherapy
- DiarrheaStartDayRelativeToD1Chemo: beginning of diarrhea relative to day 1 of chemotherapy
- DiarrheaEndDayRelativeToD1Chemo: end of diarrhea relative to day 1 of chemotherapy
- FirstCDiffDayRelativeToD1Chemo: day of the first positive test for *Clostridioides difficile* infection relative to day 1 of chemotherapy

## Technical Validation

We have previously published on both the gut microbiome and serum metabolome of these patients as interim analyses^2,4,5,25^. The material provided in this article contains all data from the entire study. For technical validation, we use the subset of patients from whom we analyzed both stool and serum samples. We perform 3 analyses to support the technical quality of data. In the first analysis, we evaluate the microbiota database. In the second analysis, we evaluate the metabolomic database. In the third analysis, we integrate the two databases.

### Alpha diversity in the gut microbiota

**Fig. 2:**
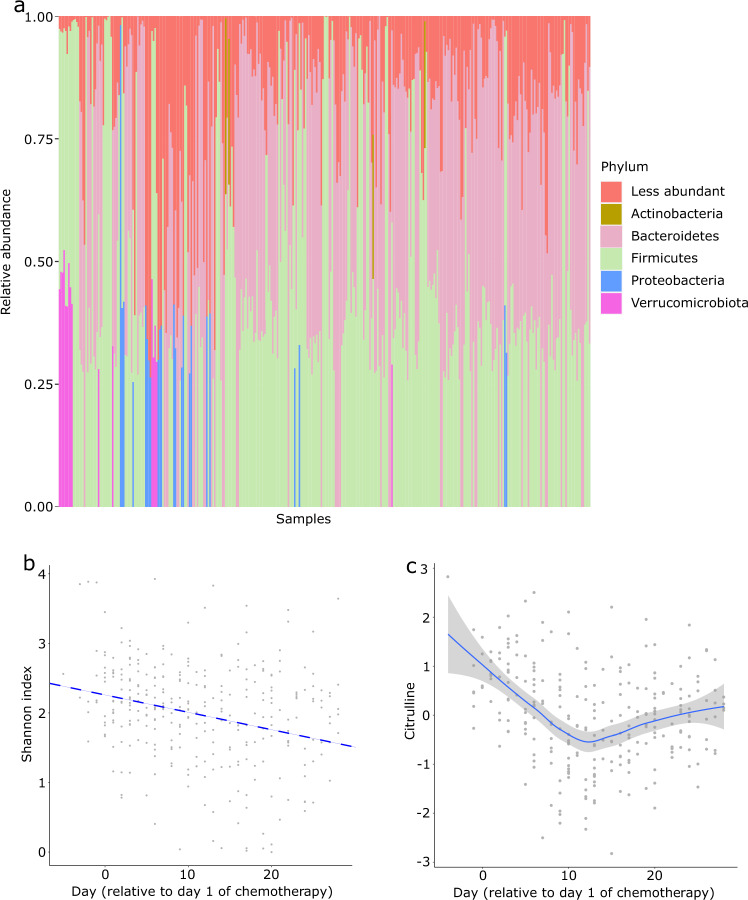
Gut microbiota taxonomic distribution, alpha diversity, and serum citrulline dynamics. (**a**) Distribution of the 5 most abundant phyla among samples. (**b**) Shannon index on the gut microbiota over time. The regression line was derived from a mixed effect model with patient ID as a random effect. (**c**) Serum citrulline levels over time. Citrulline levels are after rank-based inverse normal transformation.

The distribution of the 5 most abundant phyla among the samples is shown in Fig. 2a, with Firmicutes and Bacteroidetes being the two most abundant phyla. Previous studies have shown a decrease in alpha diversity during induction chemotherapy in AML patients^3,26,27^. We evaluated whether our dataset captures this pattern. After removing samples with < 1000 copies/mL of 16S rRNA gene or < 5000 reads, we used scaling with ranked subsampling (*SRS* package)^28^ with normalization to the lowest sequencing depth (5,021 reads) to adjust for sample depth variability. We aggregated ASVs at the genus level. Using the Shannon index^29^ to estimate alpha diversity (package *vegan*), a decline in diversity over time was apparent (Fig. 2b). To quantify this decline while accounting for the longitudinal nature of data (*i.e*., multiple timepoints per patient), we built a linear mixed effect model (*lme4* package in R) in the form of Shannon index ~ (1|patient ID) + day, where patient ID was considered a random effect and day was the sample collection day relative to day 1 of chemotherapy. After controlling for patient ID, there was a significant decline in alpha diversity over time (regression line in Fig. 1a). The regression coefficient for “day” was −0.025, with a 95% confidence interval of −0.034 to −0.017, indicating a negative slope (*p* < 0.01 from 200 bootstraps using *bootMer*).

### Citrulline dynamics in the serum metabolome

Citrulline is an amino acid produced exclusively by intestinal epithelial cells^30^, with circulating levels indicating total functioning enterocyte mass. Citrulline has been established as a biomarker for intestinal epithelial health^30–32^, with lower levels indicating intestinal epithelial cell loss. Between 10–14 days after starting mucotoxic chemotherapy, citrulline levels reach a nadir, followed by gradual recovery^31,33^. We evaluated whether our metabolomic dataset captures this pattern. Preprocessing of the metabolomic database included removing metabolites present in fewer than 50% of the samples and zero imputation using the half minimum of the observed values for each remaining metabolite. Rank-based inverse normal transformation was used for normalization. Using a loess smoother, we found a marked decline in citrulline until about day 12, after which citrulline slowly rose towards baseline (Fig. 2c). Week 2 of chemotherapy corresponds with maximal cytotoxic damage to the intestinal epithelium, thus supporting the validity of our database.

### Integration of multi-omics data

A unique feature of the present study is the multi-omics nature of data in patients with AML. Many microbial metabolites are normally found in the blood^7,8^. Examples include microbial derived metabolites of dietary tyrosine and tryptophan, with important effects on host physiology. We identified 139 same-patient pairs of serum and stool samples, with the stool sample collected within 24 hours prior to the serum sample. Using these pairs, we evaluated whether biologically meaningful connections can be found between the gut microbiota and next-day serum metabolites. We chose a 24 hour interval between samples in each pair to minimize intervening events and to account for the short half-life of many circulating metabolites. Preprocessing of the microbiota database included removing samples with < 1000 copies/mL of 16S rRNA gene or < 5000 reads, removing ASVs present in < 10% of the samples, and removing genera with a mean relative abundance < 0.005. This process yielded 33 genera for further analysis. Taxon abundances were centered log-ratio transformed to account for data compositionality^34^. Preprocessing of the metabolomic database was similar to the previous technical validation using citrulline dynamics and yielded 632 metabolites. The 33 genera were used as predictors of the 632 metabolites in sparse canonical correlation analysis (sCCA, *PMA* package, function *CCA*)^35^.

sCCA is a method to integrate multi-omics datasets with the ability to select more biologically relevant sets of features. sCCA identifies strongly associated metabolite-microbe groups by finding linear combinations of variables from each dataset maximally correlated with each other while simultaneously thresholding variable specific weights to induce sparsity and performing variable selection. This procedure applies L1-penalized matrix decomposition of the cross-product matrix similar to a LASSO regression problem^36^, thus variables are selected based on their importance to the overall microbe-metabolite covariance. As a result, taxa and metabolites with non-zero loading coefficients are those driving the overall correlation between the two datasets. Hyperparameter tuning was done through 50 permutations (*CCA.permute* function) and the best set of penalty values for each dataset were used to fit the final model. An overall correlation coefficient between the two datasets was also estimated using the correlation coefficient in the first canonical variable. The 99% confidence interval for the correlation coefficient was estimated from 1000 bootstraps. Pairwise Pearson correlation coefficients were calculated between each selected genus and metabolite and the correlogram was visualized by a heatmap (*pheatmap* package).

**Fig. 3:**
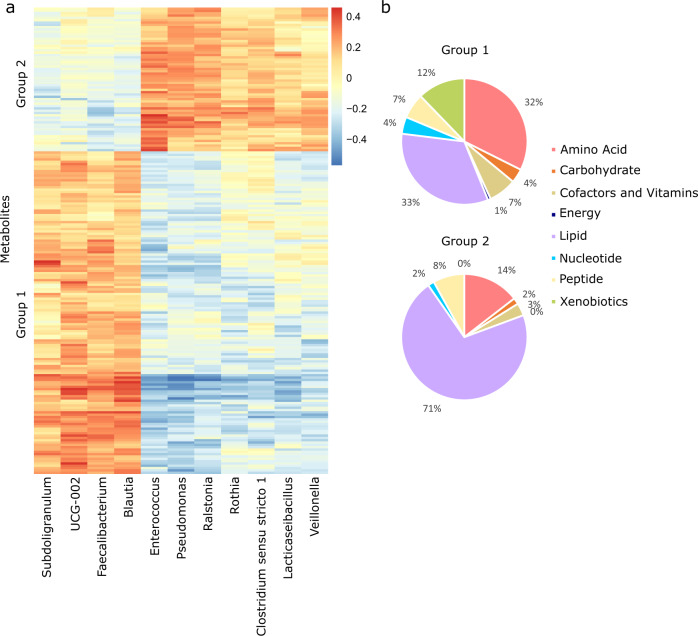
Integrated multi-omics. (**a**) Heatmap correlogram showing Pearson correlation coefficient between each final gut microbiota genus and each final serum metabolite remaining in the final results of sparse canonical correlation analysis. UCG-002 is a genus in the *Oscillospiraceae* family. (**b**) Distribution of metabolites in groups 1 and 2 in panel (a) in different metabolic pathways.

Eleven genera and 201 metabolites drove the association between the gut microbiome and serum metabolome. The overall correlation coefficient between the two datasets was 0.79 (99% confidence interval: 0.71–0.83, *p* < 0.001). The heatmap in Fig. 3a visualizes univariate correlations between these taxa and metabolites, and MicrobiomeMetabolomeHeatmap deposited in Figshare^20^ details them in tabular format. There was a clear separation of genera into two groups based on their metabolite associations. The first group contained obligate anaerobic commensal genera in the Clostridia class (*Faecalibacterium*, *Subdoligranulum*, *Blautia*, and an *Oscillospiraceae* genus UCG-002) with a plethora of beneficial effects such as butyrate production and anti-inflammatory properties^37–39^. The second group contained genera with frequently pathogenic species in patients with cancer including *Enterococcus*, *Pseudomonas*, *Rothia*, and *Veillonella*. These 2 groups showed stark differences in the metabolic pathways of their positively associated metabolites (Fig. 3b). Metabolites associated with the first group were enriched in amino acid and xenobiotic pathways, while the second group metabolites were enriched in the lipid pathway. Among metabolites in group 1 were known microbial metabolites of dietary tryptophan (indoleacetate^40^) and tyrosine (p-cresol sulfate^41^) as well as butyrate/isobutyrate.

## Usage Notes

We present repositories for a longitudinal dataset of gut microbiota and serum metabolomics from hospitalized patients with AML receiving induction chemotherapy at the University of Minnesota. These data, combined with the curated clinical metadata presented here, provide a unique opportunity for hypothesis generation. As an example, we illustrated how the microbiome and metabolome datasets can be integrated to identify novel associations for further testing in future studies. To our knowledge, this is the first public, patient- and sample-level, multi-omics database offering the interested user access to raw amplicon sequences, metabolomic data, and detailed clinical metadata in patients with AML receiving induction chemotherapy. In our multi-omics example, and by providing access to the code, we have provided a step-by-step tutorial on how sCCA may be used to integrate microbiome and metabolomics data while accounting for data compositionality and sparsity.

One limitation of this work is the lack of curated dietary data as an important determinant of both gut microbiota^42^ and serum metabolome^9,43^. In addition, although antibacterial antibiotic prophylaxis in this patient population utilizes fluoroquinolones in most centers^44^, it is not universal. Therefore, specific patterns of microbiota change may not be generalizable worldwide. Similarly, microbiome-metabolome associations found here are likely not fully generalizable to healthy individuals because of the multitude of insults to the intestinal barrier and gut microbiota in patients with AML. Finally, species-level inferences cannot be reliably made from short amplicon data^45^, a limitation that can be overcome by shotgun sequencing.

## Data Availability

The custom R code is available in Figshare^20^.
